# Circulating T cells: a promising biomarker of anti-PD-(L)1 therapy

**DOI:** 10.3389/fimmu.2024.1371559

**Published:** 2024-03-21

**Authors:** Junlei Hou, Xuezhi Yang, Shuanglong Xie, Bo Zhu, Haoran Zha

**Affiliations:** ^1^ Institute of Cancer, Xinqiao Hospital, Third Military Medical University, Chongqing, China; ^2^ Chongqing Key Laboratory of Immunotherapy, Xinqiao Hospital, Third Military Medical University, Chongqing, China; ^3^ Department of Oncology, PLA Rocket Force Characteristic Medical Center, Beijing, China

**Keywords:** circulating T cells, anti-PD-(L)1 therapy, biomarker, CD8+ T lymphocyte subsets, CD4+ T cell

## Abstract

Anti-PD-(L)1 therapy has shown great efficacy in some patients with cancer. However, a significant proportion of patients with cancer do not respond to it. Another unmet clinical need for anti-PD-(L)1 therapy is the dynamic monitoring of treatment effects. Therefore, identifying biomarkers that can stratify potential responders before PD-(L)1 treatment and timely monitoring of the efficacy of PD-(L)1 treatment are crucial in the clinical setting. The identification of biomarkers by liquid biopsy has attracted considerable attention. Among the identified biomarkers, circulating T cells are one of the most promising because of their indispensable contribution to anti-PD-(L)1 therapy. The present review aimed to thoroughly explore the potential of circulating T cells as biomarkers of anti-PD-(L)1 therapy and its advantages and limitations.

## 1 Introduction

Over the past decade, anti-PD-(L)1 therapy has revolutionized the clinical treatment of cancer. However, only some patients with cancer benefit from anti-PD-(L)1 therapy, whereas most patients eventually experience disease progression. Furthermore, a few patients receiving anti-PD-(L)1 therapy succumb to hyperprogressive diseases. Therefore, identifying biomarkers that can stratify patients who will benefit from anti-PD-(L)1 therapy is crucial in clinical practice.

The US Food and Drug Administration (FDA) has approved some biomarkers that can predict the efficacy of anti-PD-(L)1 therapy in several cancer types, including PD-L1 expression (assessed using immunohistochemistry), mismatch repair deficient/microsatellite instability-high (dMMR/MSI-high), and tumor mutation burden (TMB). These biomarkers are mostly based on tumor lesions sampled through invasive surgery or biopsy. Unfortunately, Previous studies have revealed that tumors usually exhibit high temporal and spatial heterogeneity (1), making them unrepresentative of the entire immune landscape based on a biopsy sample from a single site. For example, approximately 55% of patients with advanced non-small cell lung cancer (NSCLC) who exhibit PD-L1 expression in at least 50% of tumor cells do not benefit from the therapy (2). Therefore, there is an urgent need to develop new approaches using robust biomarkers associated with anti-PD-(L)1 therapy.

Liquid biopsy has emerged as an appealing method for identifying new biomarkers that reflect the general immune landscape (3, 4). Among the identified biomarkers, circulating T cells are one of the most promising for three reasons: 1) It has been historically accepted that effective anti-PD-(L)1 therapy relies on the reactivation of pre-existing T cells of the tumor, which exhibit high programmed cell death protein 1 (PD-1) expression levels; however, recent data suggest that effective anti-PD-(L)1 therapy relies on the continuous recruitment of new T cells from circulation (5). Accordingly, recent studies demonstrated the existence of anti-PD-(L)1 therapy-responding T cell subsets in circulation (6, 7); 2) A previous theory suggested that activated T cells infiltrate the tumor, become dysfunctional, and finally die within tumor microenvironment (TME); however, recent research indicates that some tumor-infiltrating T cells can escape from the tumor and re-enter circulation (8). indicating that some circulating T cell subsets may reflect the status of tumor-infiltrating T cells; 3) Given the gut microbiota is involved in determining the efficacy of anti-PD-(L)1 therapy (9), circulating T cells against specific ectopic bacteria may serve as a biomarker of anti-PD-(L)1 therapy. Consistently, recent research has indicated that bacteria-specific T follicular helper (Tfh) cells exist in the circulation and are correlated with PD-(L)1 efficacy (10).

This review aimed to thoroughly explore circulating T cell subsets in the context of PD-(L)1 efficacy. The advantages and limitations of the different subsets and functional states of circulating T cells were also discussed.

## 2 CD8^+^ T cell subsets as a potential biomarker

**Figure 1:**
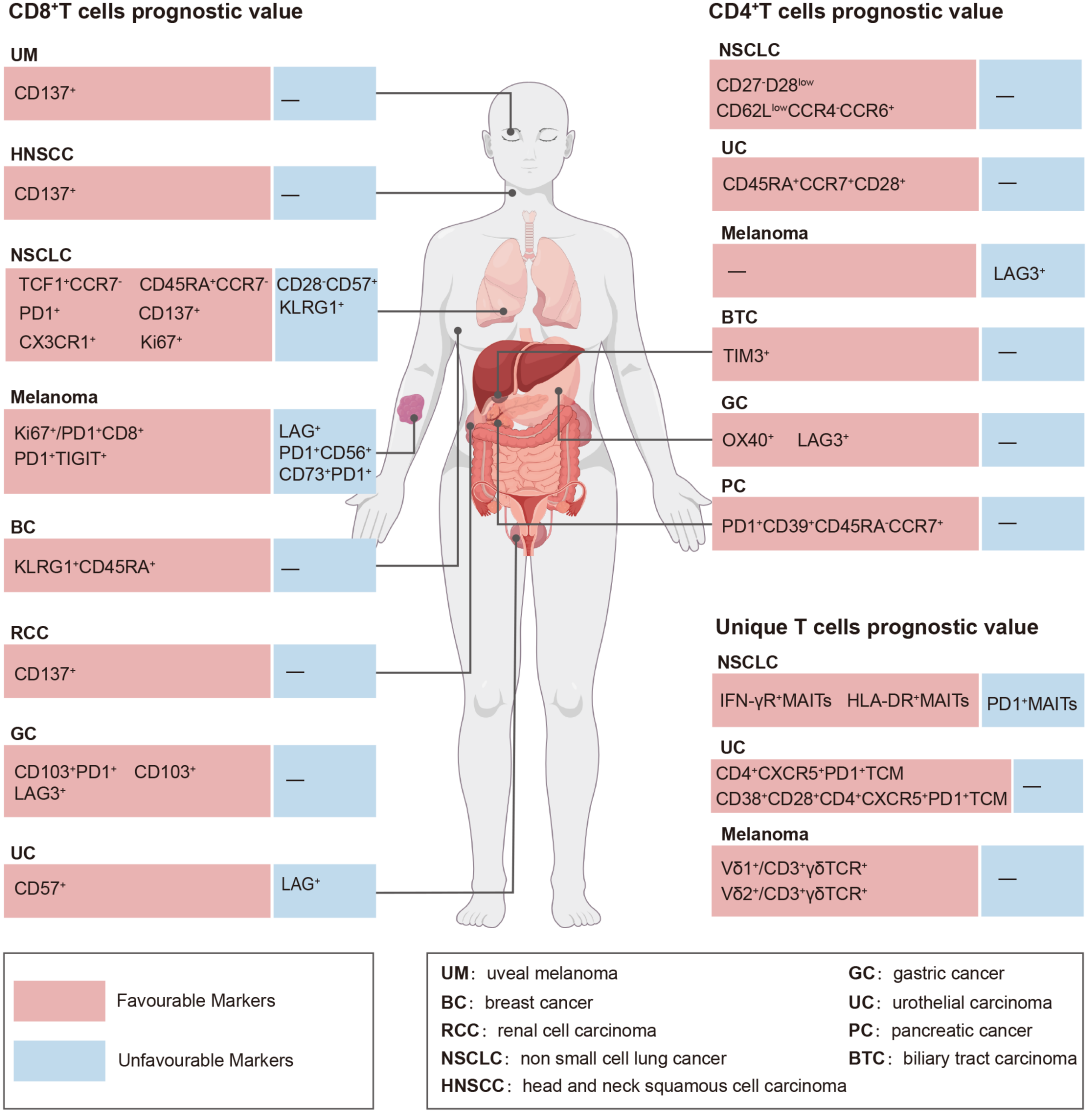
Prognostic value of circulating T cell subsets in the context of PD-(L)1 efficacy.

The number of peripheral CD8^+^ T cell subsets is closely associated with immunotherapy efficacy (11–13). Herein, we reviewed the predictive impact of peripheral blood CD8^+^ T cell subsets on efficacy before and after anti-PD-(L)1 therapy (**Table 1**, **Figure 1**).

**Table 1:** Predictive outcome of peripheral conventional CD8^+^ T cell subsets in PD-(L)1 therapy.

Subset	Sampling time	Biomarker	Method	%Biomarker of R vs NR	Cut-off	Outcome (high VS low)			Cancer type	Enrollment	Ref	
						ORR	OS (mos)	PFS (mos)				
Tem	Pre-ICB	%TCF1^+^/CCR7^-^CD8^+^	FACS	41% vs 22%	31%	-	-	17.0 vs 3.0	aNSCLC	22	(14)
		#CD28^+^CD8^+^	FACS	236 vs 138/μL	190/uL	-	NR vs. 16.2	NR vs. 8.7	NSCLC	87	(15)
	On-ICB	Δ_w3_T_IE_	FACS	10.04% vs -0.58%	0.8%	-	NR vs 9.6	-	MM	30	(16)
			FACS	3.3% vs -1.3%	0.8%	-	NR vs 4.2	-	MM	20	(16)
		#Large clones	scRNA-seq	-	Median	-	NR vs 30.7*	NR vs 5.6*	MM	69	(17)
		%TCF1^+^/CCR7^-^CD8^+^	FACS	47% vs 26%	36.5%	-	-	18.0 vs 3.5	aNSCLC	20	(14)
Terma	Pre-ICB	%CD45RA^+^CCR7^-^/CD8^+^	FACS	43.1% vs 29.7%	-	-	-	-	NSCLC	71	(18)
		%CD57^+^/CD8^+^	CyTOF	48.1% vs 14.9%	-	-	-	-	mUC	20	(19)
			CyTOF	-	25.9%	-	NR vs 4.3*	-	mUC	50	(19)
		%CD28^-^CD57^+^KLRG1^+^/CD8^+^	FACS	-	39.5%	-	3.2 vs NR	1.3 vs 1.8	aNSCLC	37	(20)
			FACS	-	39.5%	0% vs 30%	2.8 vs 20.8	1.8 vs 6.4	aNSCLC	46	(20)
			FACS	-	39.5%	-	3.1 vs 20.8	1.7 vs 3.8	aNSCLC	83	(20)
	On-ICB	CD45RA^+^CCR7^-^CD8^+^	FACS	52% vs 31%	-	-	-	-	NSCLC	71	(18)
Trm	On-ICB	%CD103^+^/PD1^+^CD8^+^	FACS	-	3.6%	-	-	↑	GC	25	(21)
		%CD103^+^_2w/pre_	FACS	-	2.8%	-	-	↑	GC	25	(21)
Tex	Pre-ICB	%LAG3^+^/CD8^+^	FACS	-	2%	-	-	2.7 vs 1.1	aGC	30	(22)
		%LAG3^+^/CD8^+^	FACS	-	-	39% vs 53%	22.2 vs 75.8	-	Melanoma	188	(23)
			FACS	-	-	20% vs 49%	12.4 vs 75.8	2.7 vs 10.6	Melanoma	76	(23)
			FACS	-	-	0% vs 49%	4.7 vs 27.5	1.2 vs 3.6	UC	94	(23)
Tex	On-ICB	%LAG3^+^/CD8^+^	FACS	-	2%	-	-	3.3 vs 1.5	aGC	30	(22)
		%(Ki67^+^/PD1^+^CD8^+^)/TB	FACS、 CyTOF	-	1.94	61% vs 10%	↑	↑	aMelanoma	23	(24)
				-	1.94	73% vs 29%	↑	-	19	19	(24)
		PD1^+^TIGIT^+^	FACS	-	17%	-	NR vs 8.26*	-	aMelanoma	26	(25)
Teff	Pre-ICB	#PD1^+^CD8^+^	FACS	-	49/uL	-	NR vs 19.5*	21 vs 2.1*	aNSCLC	31	(26)
		%CD137^+^CD8^+^/PBMC	CyTOF	45.4% vs 37.6%	0.8%		17.0 vs 5.0	11.0 vs 3.0	NSCLC	66	(27)
			CyTOF	-	0.8%	-	-	NR vs 5.0	RCC, UM HNSCC	43	(27)
		%PD1^+^/CD56^+^CD3^+^	FACS	-	16.6%	27.8% vs 59.5%	16.8 vs NR*	3.1 vs 6.5*	aMelanoma	73	(28)
		%CD73^+^ PD1^+^/CD3^+^CD8^+^	FACS	0.85% vs 3.02%	2.3%	-	6.9 vs 22.4	2.7 vs 9.0	aMelanoma	100	(29)
		KLRG1^+^CD45RA^+^/CD8^+^	FACS	37.9% vs 14.8%	-	-	-	-	HR^+^MBC	16	(30)
	On-ICB	CX3CR1 score	FACS	-	20%	80% vs 0.05%	NR vs 19.5	8.6 vs 5.7	NSCLC	36	(31)
T_Proliferat_	On-ICB	Early or delayed PD-1^+/neg^ CD8^+^ T cells response	FACS	-	4w	15.4% vs 57%	-	-	aNSCLC	27	(32)
		%Ki67_D7/D0/_PD1^+^CD8^+^	FACS	-	2.8	33.3% vs 6.3%	14.8 vs 14.2	8.7 vs 3.9	TET	31	(33)
			FACS	-	2.8	43.8% vs 11.8%	13.8 vs 2.0	6.0 vs 1.4	aNSCLC	33	(33)
			FACS	-	2.8	38.5% vs 5.0%	NR vs 7.0	10.9 vs 2.1	aNSCLC	46	(33)

T_IE_: CD3^+^CD8^+^CD45RA^−^CD45RO^high^CD27^−^CCR7^−^.

Large clones: clones with count numbers >0.5% of the total number of clones per chain as ‘large’.

CX3CR1 score: % change of CX3CR1 in CD8 ^+^ T cells from baseline.

#: Absolute number.

%: Percentage.

*: The specific values are not given in the original article, and the results are predicted by GetData software.

↑: Increased survival time in patients with higher biomarker expression.

-: Not mentioned in the article.

### 2.1 Memory CD8^+^ T cell subsets

Upon antigen stimulation, naïve T cells differentiate into T effector (Teffs) cells. After antigen elimination, most Teff cells undergo apoptosis, whereas a small proportion of Teff cells differentiate into long-lived memory T cells (34). Memory CD8^+^ T cells recirculating throughout the bloodstream can induce a rapid and robust response upon antigen reengagement, thereby playing a crucial role in sustaining long-lasting protective immunity. Circulating memory CD8^+^ T cells can be categorized into three distinct subtypes: stem central memory (Tscm), central memory (Tcm), and effector memory (Tem). Furthermore, memory CD8^+^ T cells that reside within affected tissues and exhibit limited recirculation capacity are called tissue-resident memory T (Trm) cells. Trm cells can also re-enter circulation under specific contexts (35). Current evidence indicates that memory CD8^+^ T cells in the peripheral blood correlate with responsiveness to immunotherapy, indicating their predictive value as a biomarker.

### 2.2 Effector memory CD8^+^ T cell (Tem)

Tem cells are usually characterized by a CD45RO^+^C-C motif chemokine receptor 7 (CCR7)^-^killer cell lectin-like receptor G1 (KLRG1)^high^ phenotype in patients with cancer. Tem cells emerge among early responders to immunotherapy, and their early expansion in the circulation is correlated with a durable response and improved objective response rates (16). In addition, Tem cells persist in the peripheral blood of patients with melanoma who experience durable benefits from immunotherapy (36).

### 2.3 Baseline circulating Tem cells

Tem cells have been extensively studied as predictive markers of the baseline response to anti-PD-(L)1 therapy in patients with NSCLC (14, 15). Recent studies have demonstrated that anti-PD-(L)1 therapy does not reverse the phenotype of terminally exhausted T cells but promotes the differentiation of self-renewing progenitor T cells into newly formed effector-like T cells, including memory T cells (37). T-cell factor 1 (TCF1) is an established marker of self-renewing T cells. In patients with NSCLC receiving anti-PD-(L)1 therapy, a higher proportion of TCF1-expressing T cells was observed in the circulating CD8^+^ Tem cells of patients who achieved durable clinical benefit (DCB) at baseline than in those who were resistant to treatment. Higher TCF-1 expression was associated with longer progression-free survival (PFS) (14). CD28 is a surface marker of TCF1-expressing CD8^+^ cells. Analysis of pre-treatment peripheral blood lymphocytes from 87 patients with different tumors undergoing first-line anti-PD-(L)1 therapy revealed significantly elevated circulating CD8^+^CD28^+^ T cell counts in patients who responded to treatment (median [range] counts: 236 (30–536) vs. 138 [36-460]/μL). Using 190/μL as the cut-off, patients with higher CD8^+^CD28^+^ T cell counts exhibited significantly prolonged median PFS than patients with lower counts (not reached vs. 8.7 months, p < 0.001). In addition, they had a significantly extended overall survival (OS) (not reached vs. 16.2 months, p < 0.001) (15). These findings suggest that Tem cells are strongly associated with response to PD(L)1 therapy.

### 2.4 On-treatment circulating Tem cells

In a clinical study investigating the impact of single-cycle anti-PD-(L)1 therapy on peripheral T cell dynamics in treatment-naive patients with metastatic melanoma, a significant expansion of CD27^-^CCR7^-^CD45RO^high^ CD8^+^ T cells was observed during the third week of treatment with pembrolizumab or nivolumab. An amplification ratio > 0.8% in these cells indicated a favorable prognosis with a predictive accuracy of 90% (16). These findings highlight the significant role of Tem cells in response to PD-(L)1 immunotherapy.

### 2.5 Terminally differentiated effector memory cells

Prolonged stimulation with antigens induces the progressive differentiation of Tem cells into terminally differentiated effector memory (Terma) cells, distinguished primarily by the re-emergence of CD45RA (CD45RA^+^CCR7^-^CD28^-^CD27^-^) and robust effector functionality, limited proliferative potential, and a profusion of senescence-related phenotypic traits (38). Terma cells are present in the peripheral blood of patients with different kinds of tumor (18, 19), and their presence is strongly correlated with the outcomes of PD-(L)1 immunotherapy (17, 18).

A study on patients with NSCLC receiving nivolumab reported increased numbers of CD45RA^+^CCR7^-^CD8^+^ T cells in patients who achieved a partial response (PR) at baseline and after the second or third dose (18). CD57 is a crucial marker of Terma cells (38). A study on patients with metastatic uroepithelial cancer (mUC) receiving PD-(L)1 (atezolizumab) therapy reported a remarkable prevalence of CD57 within circulating neoantigen-specific CD8^+^ T cells, particularly in individuals who responded to atezolizumab treatment, and responders to atezolizumab exhibited an increased proportion of Terma cells in the peripheral blood (19).

In contrast, some studies have suggested that circulating Terma cells are associated with an unfavorable prognosis in patients receiving PD-(L)1 immunotherapy (17, 20). A study examining the influence of immune senescence on anti-PD-(L)1 therapy in patients with advanced NSCLC found that CD28^-^CD57^+^KLRG1^+^CD8^+^ T cells (Terma-like CD8^+^ T cells) were associated with a lack of benefit from PD-(L)1 immunotherapy (20).

The ambiguity surrounding the predictive value of circulating Terma cells in immunotherapy may be attributed to variable tumor types and their clinical stages. Therefore, broadening the scope of the tumor species and increasing the sample size for further investigation on Terma cells are necessary. Terma cells are potential circulating markers for predicting the effectiveness of immunotherapy; however, further investigation is needed.

### 2.6 Tissue resident memory cells

Trm cells persist within tissues and offer rapid and effective protective immunity against pathogens and metastatic cancer cells (39). Trm cells represent a distinct subpopulation of memory cells characterized by CD103^+^CD69^+^CD49a^+^CD8^+^ expression and unique transcriptome features, including RUNX family transcription factor 3, neurogenic locus notch homolog protein, B lymphocyte-induced maturation protein-1, and basic leucine zipper ATF-like transcription factor (40). Trm cells are mainly localized within tissues; however, they can also be detected in peripheral blood (35) and play a crucial role in the early response to anti-PD-(L)1 therapy^[6^. In patients with oral cancer receiving neoadjuvant treatment with either PD-1 monotherapy or PD-1 therapy combined with cytotoxic T-lymphocyte associated protein 4 (CTLA-4) inhibitor, single-cell and T-cell receptor (TCR) sequencing revealed that both peripheral blood and tumor-infiltrating CD8^+^ T cells exhibited activation and amplification, featuring highly overlapping clone types (6). Notably, the proportion of peripheral blood KLRG1^-^PD-1^+^CD8^+^ T cells was positively associated with pathological responses in both pre-and-on-treatment groups. The findings of the study highlight the pivotal role of neoadjuvant immunotherapy in the treatment of early-stage tumors involving Trm cells. In another study involving patients with gastric cancer who underwent surgery, individuals with a higher proportion of CD103 in peripheral blood PD-1^+^CD8^+^ T cells at week 2 of immunotherapy exhibited significantly improved PFS (21).

Tem, Terma, and Trm cells aid in distinguishing patients who are more likely to benefit from PD-(L)1 immunotherapy and predict their clinical prognosis. However, owing to contradictory reported data, the significance of Tem and Terma cells as predictors needs to be interpreted with caution. Additional studies are necessary to elucidate the specific markers of circulating memory cells associated with the prognosis of patients receiving PD-(L)1 immunotherapy.

### 2.7 Exhausted (like) CD8^+^ T cells

T cell exhaustion is the specific differentiation state of T cells induced by persistent antigens and inflammatory signals, resulting in reduced effector function, diminished proliferative capacity, altered expression of multiple inhibitory receptors, and dysregulation of transcriptional mechanisms (41). Exhausted CD8^+^ T (Tex) cells exhibit high heterogeneity, which makes it challenging to define Tex cells using limited markers and molecular patterns. In the present review, Tex cells were defined using any of the following criteria: (1) expression of at least one additional co-inhibitory receptor other than PD-1, (2) high PD-1 expression levels, and (3) expression of at least one exhaustion-associated transcriptional factor, such as thymocyte selection-associated high mobility group-box (TOX), Eomesodermin (EOMES), nuclear receptor subfamily 4A, and nuclear factor of activated T cells 1. Using this category standard, we summarized the presence of heterogeneous Tex cells in the peripheral blood of patients with cancer and their predictive value in guiding the stratification of patients who may benefit from anti-PD-(L)1 therapy (22–25, 42).

### 2.8 Baseline Tex cells

Numerous studies have reported the baseline levels of Tex cell populations to predict clinical outcomes before treatment (22, 23, 42). Lymphocyte-activation gene 3 (LAG-3) is a surface inhibitory molecule highly expressed in Tex cells. In patients with gastric cancer receiving immunotherapy, a significant positive correlation was observed between LAG3^+^CD8^+^ T cells and PFS at baseline and after the initial dose (22). Another study identified a subset of LAG3^+^ T cells as an exhausted tumor-specific subpopulation that could be rejuvenated by the PD-1/PD-L1 blockade and was associated with an improved prognosis (22). Conversely, another study suggested that circulating LAG-3^+^ CD8^+^ T cells are predictive markers for identifying patients who are unlikely to benefit from PD-1 therapy. Analysis of pre-treatment blood samples from 188 patients with melanoma undergoing PD-1 therapy revealed a median survival discrepancy of > 4 years between patients with a LAG^+^ immunophenotype and those without (22.2 months vs. 75.8 months). Furthermore, in a validation cohort of 94 patients with bladder cancer treated with PD-1 therapy, those with the LAG^+^ immunophenotype demonstrated a response rate of 0. The LAG^+^CD8^+^ T cell immunotype is an independent prognostic marker (23). The noticeable discrepancy between the results of these two studies might be attributed to the patient population or methodological differences.

Patients with melanoma resistant to anti-PD-(L)1 therapy exhibited a distinct subpopulation of CD8^+^ T cells characterized by high levels of oxidative phosphorylation (OXPHOS), CD38 and CD39 expressions, and markers of exhaustion such as TOX, PD-1, and C-X-C motif chemokine ligand 13. Single-cell transcriptome analysis revealed an overlap between CD8^+^ and intratumoral CD8^+^ T cells. The study indicated that OXPHOS^+^CD8^+^ T cells among pre-treatment peripheral blood CD8^+^ T cells correlate with immune checkpoint inhibitor resistance in patients with malignant melanoma (42).

### 2.9 On-treatment Tex cells

Changes in Tex cells after treatment can predict clinical outcomes because the systemic response to anti-PD-(L)1 therapy is dynamic and complex (43). Notably, several studies have investigated the role of post-treatment peripheral blood Tex cells in predicting the clinical outcome of anti-PD-(L)1 therapy (24, 25, 42).

In patients with stage IV melanoma receiving PD-1 (pembrolizumab) therapy, the magnitude of reinvigoration of circulating Tex cells, determined in relation to the pre-treatment tumor burden, correlated with the clinical response (24). The reinvigoration of circulating Tex cells is characterized by the expression of CD45A^lo^CD27^hi^Eomes^hi^T-bet^lo^ and co-expressed suppressor molecules (PD1, CTLA-4, and CD244), which correspond to the Tex cells’ characteristics (24). Immunoglobulin, immunoreceptor tyrosine-based inhibitory motif domains of T cell immunoreceptors, and PD1 double-positive T cells (DPOS) are present in the peripheral blood of patients with cancer and can serve as markers for predicting response to anti-PD-(L)1 therapy. In three cohorts of patients with cancer undergoing PD-1 therapy, a higher proportion of DPOS T cells in the peripheral blood after 1 month of treatment correlated with improved clinical response and extended OS (25).

In summary, peripheral circulating Tex cells play a crucial role in the response to PD-(L)1 treatment and can potentially serve as biomarkers for predicting the efficacy of anti-PD-(L)1 therapy.

### 2.10 Effector CD8^+^ T cells

CD8^+^ Teff cells express chemokine receptors, including CCR5, C-X-C motif chemokine receptor 3, and C-X3-C motif chemokine receptor 1 (44, 45), enabling their migration and infiltration from peripheral blood into the tumor microenvironment, where they exert their cytotoxic effects (45). Previous studies have shown that tumor-reactive T cells, including neoantigen-specific T cells, are present in the peripheral blood of patients with cancer and are enriched within the population of PD1^+^CD8^+^ T cells (46–48). Peripheral effector PD1^low^CD8^+^ T cells exhibit an “effector-like” phenotype (32). Current evidence has revealed the response of peripheral circulating CD8^+^ Teff cells in patients with tumors to anti-PD-(L)1 therapy and their value in predicting the prognosis of patients with cancer (15, 26, 28, 29, 31, 49).

### 2.11 Baseline Teff cells

The predictive ability of circulating Teff cells before anti-PD-(L)1 therapy has been investigated in various tumor types, including malignant melanoma, NSCLC, and gastric cancer (26, 28, 29). One study reported that among patients with advanced NSCLC receiving nivolumab treatment, treatment responders exhibited approximately two-fold higher baseline levels of PD1^+^CD8^+^ T cells in the peripheral blood than non-responders. Increased levels of circulating PD1^+^CD8^+^ T cells are associated with prolonged OS and PFS (26). In another clinical cohort study involving metastatic tumors of various origins, a higher abundance of baseline CD137^+^CD8^+^ T cells was observed in the peripheral blood of patients who responded to anti-PD-(L)1 therapy. Elevated levels of CD137^+^CD8^+^ T cells in peripheral blood are associated with improved PFS and OS in patients (27). The CD137 receptor (4-1BB, tumor necrosis factor receptor [TNFR] superfamily) belongs to the costimulatory TNFR family and is expressed on activated CD8^+^ T cells (50). Similar results have been observed in patients with hormone receptor-positive metastatic breast cancer treated with the cyclin-dependent kinase 4/6 inhibitor (palbociclib) and PD-1 therapy (pembrolizumab) (30).

In contrast, certain observations have been made for specific subpopulations of PD1^+^ Teff cells. In patients with melanoma, the proportion of PD-1^+^CD56^+^ T cells in the peripheral blood is inversely correlated with clinical benefit (28). Another study reported a correlation between an elevated number of CD73^+^PD1^+^CD8^+^ T cells in the peripheral blood and an unfavorable response to anti-PD-(L)1 therapy (29). Among patients with advanced melanoma receiving nivolumab treatment, those who experienced clinical benefits exhibited considerably lower baseline proportions of circulating CD73^+^PD1^+^CD8^+^ T cells than non-responding patients (29).

### 2.12 On-treatment Teff cells

Studies have shown that Tex cells within the tumor are replaced by CD8^+^ Teff cells recruited from the peripheral blood. This suggests that peripheral circulating T cells respond to anti-PD-(L)1 therapy (5). Circulating Teff cells are early responders to anti-PD-(L)1 therapy, and changes in circulating Teff cells caused by PD-(L)1 treatment strongly correlate with the prognosis of patients with tumor (31, 49).

A clinical trial investigating the impact of anti-PD-(L)1 therapy on CD8^+^ T cell function utilized single-cell sequencing of peripheral blood samples obtained from patients with melanoma undergoing immunotherapy. The results revealed the upregulation of natural killer cell granule protein-7 in CD8^+^ Teff cells in responders, whereas its downregulation was observed in non-responders (49). CX3CR1 belongs to a class of chemokine receptors that are highly expressed on the surface of Teff cells (45). Studies have shown that an elevated proportion of CX3CR1^+^ subpopulations within circulating CD8^+^ T cells at an early stage following anti-PD-1 therapy correlates with a favorable response and improved survival in patients with NSCLC (31). Furthermore, another study revealed an increase in neoantigen-reactive T cells among responders to PD-L1 therapy (51). However, this potential was constrained by the limited number of pre-and post-treatment sample pairs. Furthermore, the identification of tumor antigen-specific CD8^+^ T cells remains limited, and their universality necessitates further investigation.

### 2.13 Proliferating CD8^+^ T cells

Reinvigoration of pre-existing tumor-infiltrating T cells by anti-PD-(L)1 therapy is insufficient to inhibit tumor growth. Maximizing anti-PD-(L)1 therapy-mediated tumor control requires newly recruited T cells from the circulation (5). In patients with chronic infection and cancer, the TCF1-expressing subset of CD8^+^ T cells is responsible for the anti-PD-(L)1 therapy-driven T cell proliferation burst, which depends on CD28 signaling (52–54). Notably, most TCF1^+^CD8^+^ T cells in individuals with cancer reside in tumor-draining lymph nodes (55, 56). Under anti-PD-(L)1 therapy, TCF1^+^CD8^+^ T cells tend to proliferate (56). Subsequently, proliferating CD8^+^ T cells are released into the bloodstream, as evidenced by the increased proportion of Ki67^+^ CD8^+^ T cells in the blood after PD-1 therapy (53). The expansion of T cells in the peripheral blood of patients with cancer consistently predicts a better clinical response to anti-PD-L1 therapy (57). Numerous studies have consistently reported that Ki-67 expression in peripheral blood CD8^+^ T cell subsets exhibits a transient increase solely during the initial cycle after immunotherapy (32, 33). Therefore, the independent predictive and prognostic value of Ki-67-expressing CD8^+^ T cells as biomarkers of anti-PD-(L)1 therapy remains debatable.

A longitudinal analysis of blood samples from patients with advanced NSCLC undergoing PD-1 therapy revealed that approximately 70% of the patients exhibited an increased proportion of Ki67^+^PD-1^+^CD8^+^ T cells after the initial or second treatment cycle (32). This suggests that PD-1 therapy stimulates the proliferation of peripheral circulating PD1^+^CD8^+^ T cells. Among patients who experienced clinical benefits, 80% demonstrated a PD-1^+^CD8^+^ T-cell response within 4 weeks of treatment. PD-1 therapy-driven T cell proliferation is not uniform across different T cell subsets, as researchers found that Epstein–Barr virus-specific CD8^+^ T cells exhibit a diminished response to PD-1 therapy, indicating that the responsive cells might be specific to the tumor (32). Proliferating CD8^+^ T cells exhibit the characteristics of an effector-like phenotype (Human leukocyte antigen-DR^+^, CD38^+^, and B-cell lymphoma 2^lo^) (32). Comparable outcomes have been observed in patients with mUC treated with PD-L1 therapy. One study reported that within the identified neoantigen-reactive CD8^+^ T cells (NART), patients with disease control exhibited a Ki67^+^PD1^+^ effector phenotype in peripheral NART, indicating that the early amplification and activation of effector NART in patients with mUC is associated with a positive response to anti-PD-(L)1 therapy (58). Another study reported that an early proliferative response of PD1^+^CD8^+^ T cells was correlated with improved clinical outcomes. Among patients with thymic epithelial tumors and NSCLC undergoing PD-1 therapy, peripheral blood PD-1^+^CD8^+^ T cells (Ki-67_D7/D0_) exhibited a proliferative response within the initial week of treatment, enabling the differentiation between response to therapy and disease progression. This finding was confirmed in patients with NSCLC receiving PD-1 therapy. Tumor-specific CD8^+^ T cells exhibited a significant increase in Ki-67 expression on day 7, whereas virus-specific CD8^+^ T cells did not, indicating the specificity of PD-1 therapy for expanding tumor-reactive CD8^+^ T cells (33).

In summary, anti-PD-(L)1 therapy effectively restored the expansion of peripheral circulating tumor-reactive CD8^+^ T cells. The proliferation of peripheral circulating tumor-reactive CD8^+^ T cells has been associated with improved clinical outcomes and prolonged survival. However, additional studies are required to establish the optimal sampling time due to the highly dynamic expression of Ki-67 in circulating CD8^+^ T cells.

## 3 CD4^+^ T cell subsets as a potential biomarker

Similar to CD8^+^ T cells, CD4^+^ T cells, including naïve, Tcm, Tem, and Terma CD4^+^ T cell subsets and a unique group of regulatory CD4^+^ T cells (Tregs), characterized by CD25 and Forkhead box protein P3 expressions, are abundantly present in the peripheral blood. It is generally accepted that CD8^+^ T cells play a critical role in anti-PD-(L)1 therapy, whereas the importance of CD4^+^ T cells is underappreciated. However, few studies have revealed the significance of circulating CD4^+^ T cell subsets in predicting the efficacy of anti-PD-(L)1 therapy. Herein, we summarized the predictive value of CD4^+^ T cell subsets in patients with cancer treated with PD-(L)1 (**Table 2**, **Figure 1**).

**Table 2:** Predictive outcome of peripheral CD4^+^ T cell subsets in PD-(L)1 therapy.

Subset	Sampling time	Biomarker	Method	%Biomarker of R vs NR	Cut-off	Outcome (high VS low)			Cancer type	Enrollment	Ref
						ORR	OS (mos)	PFS (mos)			
Naive	Pre-ICB	%CD45RA^+^CCR7^+^ CD28^+^/CD4^+^	FACS	42.6%* vs 22.6%*	-	-	-	31.7 vs 7.9	UC	22	(59)
Tcm	Pre-ICB	%PD1^+^CD39^+^/CD45RA^-^CCR7^+^CD4^+^	CyTOF	9.5%* vs 7.5%*	-	-	-	20.6* vs 10.2*	aPC	34	(60)
		%CD27^-^CD28^low^/CD4^+^	FACS	-	40%	44.8% vs 0%	-	23.7 vs 6.1	NSCLC	51	(61)
Tem	Pre-ICB	%PD1^+^CD39^+^/CD45RA^-^CCR7^-^CD4^+^	CyTOF	27.2%* vs 18.4%*	-	-	-	23.9* vs 10.2*	aPC	34	(60)
		%CD62L^low^ CCR4^-^CCR6^+^/CD4^+^	FACS	-	4.39%	-	NR vs 11.1	10.3 vs 4.3	aNSCLC	31	(62)
	On-ICB	%OX40^+^/CD4^+^	FACS	-	15%	-	-	3.17 vs 1.7	aGC	30	(22)
Temra	Pre-ICB	%LAG3^+^/CD4^+^	FACS	0.08%* vs 0.24%*	-	-	-	-	Melanoma	25	(63)
		%LAG3^+^/CD4^+^	FACS	-	3%	-	-	2.77 vs 1.27	aGC	30	(22)
	On-ICB	TIM3^+^CD4^+^ fold change	FACS	-	1.26	-	12.9 vs 6.6	-	BTC	77	(64)
		%LAG3^+^/CD4^+^	FACS	-	3%	-	-	3.03 vs 1.4	aGC	30	(22)
Treg	Pre-ICB	prediction formula	FACS	-	192	-	-	10.5 vs 1.7*	NSCLC	86	(65)

%: Percentage.

*: The specific values are not given in the original article, and the results are predicted by GetData software.

-: Not mentioned in the article.

### 3.1 Naïve and memory CD4^+^ T cells

Naïve CD4^+^ T cells are characterized by CD45RA^+^ and CCR7^+^ expressions, whereas CD4^+^ Tcm cell subsets are characterized by CD45RA^-^ and CCR7^+^ expressions. CD4^+^ Tcm cell subsets are T cells with long-term persistence. In a study involving 26 patients with bladder cancer who received PD-(L)1 (durvalumab) therapy, a group of naïve (CD45RA^hi/int^ CCR7^+^CD28^+^) CD4^+^ T cells from pre-treatment (week 1) time points were enriched in the peripheral blood of responders but not in that of progressors (59). In addition, the pre-treatment proportions of circulating CD4^+^ Tcm cells were associated with prolonged survival after treatment with nivolumab combined with chemotherapy in patients with metastatic pancreatic cancer (60).

### 3.2 Effector-like or exhausted-like CD4^+^ T cells

OX40 is a vital co-stimulator molecule (66). The proportion of baseline OX40-expressing circulating CD4^+^ T cells has been studied as a predictive marker of response to PD-1 therapy in patients with advanced solid tumors. CD4^+^PD1^+^OX40^+^ and CD4^+^α4β7^+^ cells among total CD4^+^ T cells have been reported to be present in higher proportions in patients with DCB, PR, or SD ≥ 6 months than in those without DCB (67). In addition to baseline level, circulating OX40^+^ CD4^+^ T cell/CD4^+^ T cell ≥ 15% was associated with better PFS in patients with advanced gastric cancer after treatment with nivolumab (22). In two independent phase I clinical trials, 19 patients with hormone receptor-positive metastatic breast cancer were treated with pembrolizumab. A study reported that the baseline proportion of circulating KLRG1^+^inducible T cell costimulator ^+^CD4^+^ T cells was significantly higher in responders than in non-responders (22.6% vs. 8.7%) (30). Another study reported that the proportion of pre-treatment PD-1^+^CD39^+^CD4^+^ Tem cells was associated with > 1-year survival (60). Horimoto et al. recently suggested that the peripheral blood of patients with NSCLC receiving PD-1 therapy contains abundant CCR4^-^CCR6^+^ CD4^+^ T cells (Th7R cells). Patients with a higher proportion of baseline Th7R cells among CD4^+^ T cells (> 4.39%) showed significantly prolonged OS and PFS (62). In addition, a subgroup of CD27^-^CD28^low^ cells, known as highly differentiated T cells, appears to be associated with the prognosis of anti-PD-(L)1 therapy (68).

### 3.3 Terminally differentiated effector memory CD4^+^ T Cells

CD4^+^ Terma cells (characterized by CD45RA^+^ and CCR7^-^ expression) represent a group of terminally differentiated T cells with downregulation of costimulatory molecules and upregulation of inhibitory molecules. The proportions of CD38^+^CD39^+^CD127^-^GARP^-^ CD4^+^ Terma cells were higher in the peripheral blood of patients with recurrence than in those without recurrence after treatment with nivolumab plus ipilimumab (69). In addition, an increased population of CD4^+^T cell immunoglobulin and mucin domain-containing protein 3 (TIM3^+^)T cells after dual mitogen-activated protein kinase kinase/PD-L1 inhibition correlated with worse OS (64). In contrast to TIM3, which predicts a poor prognosis, the relationship between LAG3^+^CD4^+^ T cells and prognosis is more variable. At baseline, a high proportion of LAG3-expressing CD4^+^ T cells indicates resistance to ipilimumab plus nivolumab treatment (63). In contrast, another study reported that patients with higher proportions of LAG3^+^CD4^+^ T cells at baseline (> 3%) and after the first administration(>3%) of nivolumab had longer PFS (22).

### 3.4 Regulatory T cells

Current evidence indicates that regulatory T cells (Tregs) are essential for predicting the efficacy of anti-PD-(L)1 therapy. Notably, most studies have shown that Tregs are negatively correlated with prognosis, which is consistent with general beliefs. At baseline, the proportion of Tregs among CD4^+^ T cells was higher in patients with progressive disease (70). Furthermore, the expression of ki67 on Tregs is higher in patients with progressive disease (PD) (71). In addition, the percentage of circulating Tregs is significantly higher in patients resistant to nivolumab (65).

## 4 Unique T cell subsets

The predictive capability of immunotherapy has traditionally focused on classical CD8^+^ and CD4^+^ T cells; however, there is emerging evidence that some unique T cell subsets, including natural killer T cells (NKT), mucosa-associated invariant T cells (MAIT) (72), γδ T cells (73), CD8^-^CD4^-^T cells (double-negative T [DNT]) (74), and Tfh cells (10) can effectively identify individuals who will benefit from immunotherapy. Herein, we reviewed the current research on the role of these unique T cells in predicting immunotherapy outcomes and explored the need for further investigation into their significance in determining the predictive value of immunotherapy (**Table 3**, **Figure 1**).

**Table 3:** Predictive outcome of peripheral unique T cell subsets in PD-(L)1 therapy.

Subset	Sampling time	Biomarker	Method	%Biomarker of R vs NR	Cut-off	Outcome (high VS low)			Cancer type	Enrollment	Ref
						ORR	OS (mos)	PFS (mos)			
MAIT	Pre-ICB	%IFN-γ^+^/ MAITs	FACS	35.95% vs 13.05%	-	-	-	-	NSCLC	27	(72)
		%HLA-DR^+^/ MAITs	FACS	18.8% vs 10.5%	-	-	-	-	NSCLC	29	(72)
		%PD-1^+^/MAITs	FACS	2.52% vs 5.35%	-	-	-	-	NSCLC	46	(72)
γδT	Pre-ICB	%Vδ1^+^/CD3^+^γδTCR^+^	FACS	-	30%	-	8.0 vs. 13	-	melanoma	109	(73)
		%Vδ2^+^/CD3^+^γδTCR^+^	FACS	-	39%		14 vs. 7.0		melanoma	109	(73)
Tfh	Pre-ICB	%CD4^+^CXCR5^+^PD1^+^TCM/CD4^+^	CyTOF	-	3.06%	-	-	28.2 vs 21.1	MIBC	34	(10)
		%CD38^+^CD28^+^CD4^+^CXCR5^+^PD1^+^TCM /CD4^+^	CyTOF	-	0.43%	-	-	NR vs 23.9	MIBC	34	(10)

%: Percentage.

-, Not mentioned in the article.

### 4.1 Mucosa-associated invariant T cells (MAIT)

MAIT cells, an atypical subset of T lymphocytes, are widely distributed throughout the human body and have been implicated in the pathogenesis of various human malignancies (75, 76). While predominantly located in mucosal tissues, MAIT cells also exist in the peripheral blood, lymphoid tissue, and organs including the liver (77). These cells express a conserved alpha chain (Vα7.2-Jα33) and a restricted range of beta chains (Vβ2 or Vβ13 in humans), facilitating the recognition of vitamin B metabolite antigens presented by the major histocompatibility complex (MHC)-I-related molecule (78). Among patients with NSCLC, those who responded to PD-1 therapy exhibited a higher proportion of circulating MAIT cells expressing the IFN-γ receptor (IFN-γR) in CD3^+^ T cells. Conversely, a lower proportion of MAIT cells expressed PD-1 (72). While these findings suggest that specific subsets of circulating MAIT cells expressing interferon (IFN)-γ receptor (MAIT-IFNGR cells) may serve as promising predictive markers for therapeutic response, MAIT cells expressing interleukin-17A (MAIT-IL17 cells) might indicate potential resistance to PD-1 therapy.

### 4.2 γδ T cells

γδ T cells, a subset of unconventional T cells that operate independently of the conventional MHC restriction, constitute 1%–5% of the circulating T cell population and exhibit natural and adaptive immunity features (79). In patients with melanoma undergoing ipilimumab treatment, investigators observed that a decreased frequency of γδ T cells and an increased frequency of γδ T cells in the peripheral blood before treatment correlated with extended OS (73).

### 4.3 CD8^-^CD4^-^T cells (DNT) and NKT cells

DNT are a unique subpopulation of CD3^+^ cells that express αβ or γδ T cell receptors but lack mature surface T cell markers, including CD4, CD8, and CD56 (80). These cells are thought to play a vital role in maintaining immune system homeostasis by eliminating self-reactive immune cells and modulating the allogeneic responses (81–83). In a prior study, it was observed that patients who responded to treatment exhibited reduced DNT and elevated NKT cell counts in their peripheral blood after undergoing treatment (74).

### 4.4 Follicular helper T cells

Tfh constitute a specialized subset of CD4^+^ Teff cells that play a vital role in germinal center responses, B-cell affinity maturation, and the orchestration of lymph node structure development (84, 85). In the context of Muscle-Invasive Bladder Cancer (MIBC), patients with a high proportion of circulating Tfh cells at baseline exhibited improved prognostic survival. Mechanistically, neoadjuvant therapy proves particularly beneficial for patients with MIBC who possess pre-existing circulating Tfh cells, as these cells play a pivotal role in orchestrating the development and maturation of tertiary lymphoid structures (TLS) like cells and in promoting the infiltration of CD8^+^ T cells into the tumor microenvironment (10).

## 5 Discussion

Driven by chronic stimulation of tumor antigens, inflammation and hypoxic TME, tumor infiltrating T cells undergoes exhaustion, characterized by hierarchical loss of cytokine secretion and proliferation potential, while increase expression of various inhibitory receptors (86). Among the inhibitory receptors, PD-1 is the most attractive targets, due to its blockade refers to a significant success in cancer treatment (87). PD-1 was first cloned by Honjo et al. in 1992 (88). T cells are the major source of PD-1 in TME, while other types of cell also express it (89). PD-1 has two major ligands: PD-L1 and PD-L2 (87). PD-L1 is broadly expressed, while PD-L2 expression is restricted to hematopoietic cells, including dendritic cells, macrophages and B cells. Given the therapeutic efficacy of anti-PD-1 and anti-PD-L1 therapies are comparable in human, PD-L2 is probably of little importance in this context (87). Therefore, our review mainly focus on PD-1 and PD-L1. However, some recent studies suggest that PD-L2 are also promising therapeutic target (90, 91).

The advent of anti-PD-(L)1 therapy has greatly transformed the approach and prognosis of patients with cancer. Despite its notable impact, the response rate of anti-PD-(L)1 therapy remains low when applied in unselected patients with cancer. So far, the US FDA has approved three biomarkers that can predict the efficacy of anti-PD-1 therapy in several cancer types, including PD-L1 expression, dMMR/MSI-high, and TMB. These biomarkers are mostly based on tumor lesions sampled through invasive surgery or biopsy. Taking PD-L1 as an example, despite widespread use in clinic, PD-L1 expression as a biomarker presents limits due to technical and biological reasons, and its difficulty of assessment for pathologists (92, 93). In addition, the intra- and inter-tumor heterogeneity is another major issue for using PD-L1 as a biomarker (1, 94). Thus, the PD-L1 expression can be underestimated in small biopsies (such as bronchial and transthoracic biopsies), which are not representative of the entire tumor.

Circulating T cells has emerged as a promising biomarker that reflect the general immune landscape. Circulating T cells contains several T cell population, including memory (like) T cells, exhausted (like) T cells, effector T cells and proliferating T cells. Rencent studies suggest that anti-PD-(L)1 relies on the activation of memory (like) T cells or exhausted precursors T cells (52, 56), while activation of terminally exhausted T cells may promotes tumor progression by modulating cancer stem cells (95). Accordingly, existence of abundant number of memory (like) T cells in circulation was reported to be a favorable biomarker in patients receiving anti-PD-(L)1 therapy (14). Although numerous studies have explored the potential of circulating T cell subsets in predicting the efficacy of anti-PD-(L)1 therapy, a consensus is yet to be reached regarding the specific population and subsets of circulating T cell subsets. Consequently, prospective studies of large cohorts are urgently needed to validate the value of circulating T cell subsets in therapeutic decision-making. Lastly, it is important to note that circulating T cells are modifiable, which means a patient could been possiblely transformed toward an PD-(L)1-sensitive state before the initiation of therapy. For example, circulating bacteria-specific Tfh cells are correlated with PD-(L)1 efficacy (10). Accordingly, a recent study suggest that oral administration of bacteria or faecal microbiota transplantation were able to enhance efficacy of anti-PD-(L)1 threarpy in mice (61). Circulating T cells is a promising biomarker of PD-(L)1 therapy, as medicine becomes increasingly personalized, they may play a vital role in informing treatment decisions in the future.

## Author contributions

JH: Writing – original draft. XY: Writing – original draft. SX: Writing – original draft. ZB: Conceptualization, Writing – review & editing. HZ: Conceptualization, Writing – original draft, Writing – review & editing.
